# Bilateral vallecular cysts^☆^

**DOI:** 10.1016/j.bjorl.2016.10.003

**Published:** 2016-11-05

**Authors:** Lucas Spina, Vinícius Zanin Martins, Julio Defaveri, Regina Helena Garcia Martins

**Affiliations:** a Universidade Estadual Paulista Júlio de Mesquita Filho (UNESP), Faculdade de Medicina de Botucatu, Departamento de Oftalmologia e Otorrinolaringologia, Botucatu, SP, Brazil; b Universidade Estadual Paulista Júlio de Mesquita Filho (UNESP), Faculdade de Medicina de Botucatu, Departamento de Patologia, Botucatu, SP, Brazil

## Introduction

Laryngeal cysts are rare benign lesions, which can be asymptomatic or present with symptoms of dysphagia, dysphonia, sensation of a foreign body in the throat, and stridor. In many cases they are identified during endoscopic examination of the airways or digestive system. They are classified as ductal (retention or mucosal cysts), corresponding to 25% of the cases, or saccular (cysts that protrude from the ventricle), corresponding to 75% of the cases. This classification is based on histological features, content and site.^1^ The entire larynx can harbor cysts, the most common sites being, in order of frequency, the vocal folds, epiglottis, and valeculla,^2^ where 10% of the cases occur.^3^

## Case report

We present a case of a 54-year-old male patient, previously diagnosed with Paracoccidioidomycosis (PCM), confirmed by biopsy of a skin lesion in the malar region. In medical record review we identified an otolaryngology evaluation held five years ago in which there were no reports of respiratory, digestive or vocal symptoms and the ENT examination was normal. Among the comorbidities, the patient presented acute arterial obstruction, being treated with anticoagulant. Death occurred at age 54, the primary cause being PCM, followed by septic shock and pneumonia. The autopsy reveals PCM compromising extensively the lungs and adrenal glands.

**Figure 1:**
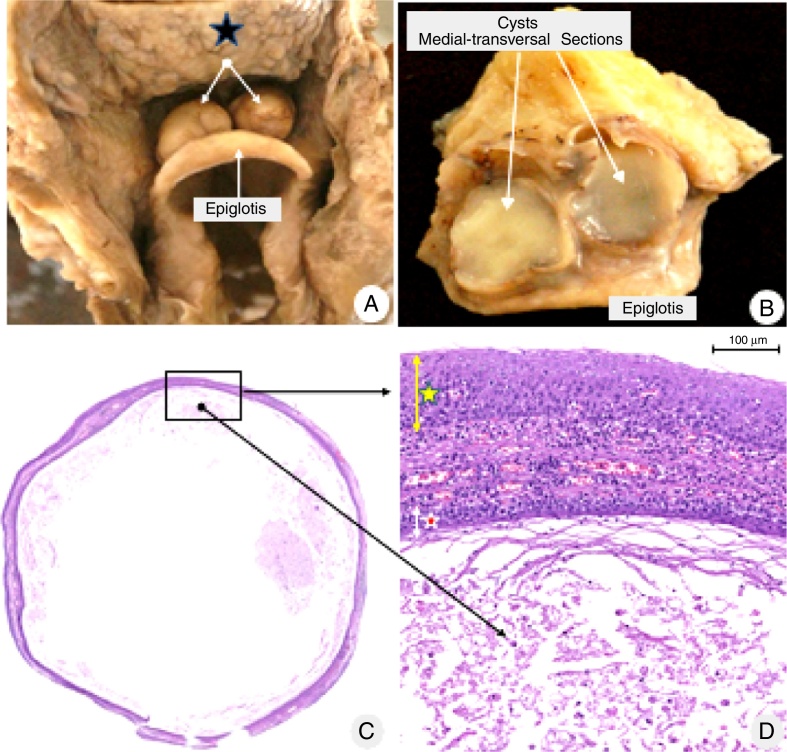
(A) Bilateral vallecular cysts (white arrows), located between the base of the tongue (blue star) and epiglottis. (B) Transversal sections of both cysts with similar macro and microscopic aspects: thin walls and filled with a mucinous yellow-tan material. (C) Panoramic microscopic section from the right cyst: thin membranous-like wall, partially filled with cellular debris and amorphous secretion (hematoxylin–eosin staining). (D) High power magnification of the demarcated area in (C). Both sides of the cysts (the outer surface indicated by a yellow star, and the inner surface by a red star) are lined by stratified, non-keratinized epithelia. The walls of both cysts are thin, delicate, composed by fibrous soft tissue, small vessels, and scarce lymphoplasmacytic infiltrate (HE staining).

The oral, pharyngeal, and laryngeal mucosa were normal at the autopsy, but two bilateral, symmetrical, domed-shape cysts (Fig. 1A), ranging from 1.5 cm to 1.8 cm in diameter was found in the vallecular region. The cysts had a tense surface and were filled with a mucinous yellow-tan material (Fig. 1B). The microscopic structure of the cysts is shown in Fig. 1C and D.

This project was approved by the Human Research Ethics Committee of our institution.

## Discussion

Vocal cysts correspond to 4.3–6% of all benign laryngeal lesions.^3^ They are more frequent in men in the fifth decade of life but can be diagnosed at all ages.^4^, ^5^ The patient presented in this study was asymptomatic; however some authors had described important symptoms as dysphagia,^3^ stridor and dyspnea.^1^ Therefore, we highlighted the importance of the endoscopic exam in patients with these symptoms.

In this case report, the cysts had a stratified, non-keratinized malpighian epithelia lining, suggestive of ductal origin for both cysts and the presence of inflammatory infiltrate in the corium indicated inflammatory origin. Although the autopsy has not identified lesions of Paracoccidioidomycosis in airway mucosa, we believe that the chronic inflammation of the mucosal, caused by this disease, allied to smoking, can result in gland obstructions and cysts, as identified in this case report.

Among the treatment methods for laryngeal cysts there is complete removal by marsupialization or excision with CO_2_ laser or electrocautery. Content aspiration alone tends to result in recurrence and is not a recommended method.^5^

## Conclusion

We presented a rare case report of a patient with bilateral vallecular cysts without digestive or respiratory symptoms. However, cysts can become large and cause stridor and dyspnea. Therefore, we highlighted the importance of the endoscopic exam in patients with these symptoms and the complete removal of the cyst for the definitive treatment.

## Conflicts of interest

The authors declare no conflicts of interest.

## References

[1] De A., Don D.M., Magee W., Ward S.L. (2013). Vallecular cyst as a cause of obstructive sleep apnea in an infant. J Clin Sleep Med.

[2] Yilmaz M., Haciyev Y., Mamanov M., Cansiz H., Yilmaz R. (2011). Epidermal inclusion cyst of the larynx. J Craniofac Surg.

[3] Romack J.J., Olsen S.M., Koch C.A., Ekbom D.C. (2010). Bilateral vallecular cysts as a cause of dysphagia: case report and literature review. Int J Otolaryngol.

[4] Sataloff J.B., Defatta R.A., Hawkshaw M.J., Sataloff R.T. (2012). Ventricular cyst of the larynx. Ear Nose Throat J.

[5] Young V.N., Smith L.J. (2012). Saccular cysts: a current review of characteristics and management. Laryngoscope.

